# Blood Coagulation Activities and Influence on DNA Condition of Alginate—Calcium Composites Prepared by Freeze-Drying Technique

**DOI:** 10.3390/md22090415

**Published:** 2024-09-10

**Authors:** Małgorzata Świerczyńska, Paulina Król, César I. Hernández Vázquez, Klaudia Piekarska, Katarzyna Woźniak, Michał Juszczak, Zdzisława Mrozińska, Marcin H. Kudzin

**Affiliations:** 1 Łukasiewicz Research Network, Lodz Institute of Technology, Marii Sklodowskiej-Curie 19/27, 90-570 Lodz, Poland; 2 Institute of Polymer and Dye Technology, Faculty of Chemistry, Lodz University of Technology, Stefanowskiego 16, 90-537 Lodz, Poland; 3 Department of Molecular Genetics, Faculty of Biology and Environmental Protection, University of Lodz, Pomorska 141/143, 90-236 Lodz, Poland

**Keywords:** alginate, calcium, composite, polymer functionalization, blood coagulation, activated partial thromboplastin time (aPTT), prothrombin time (PT), plasmid relaxation assay

## Abstract

The aim of this research was to synthesize and characterize alginate–calcium composites using a freeze-drying method, with a focus on their potential applications in biomedicine. This study specifically explored the biochemical properties of these composites, emphasizing their role in blood coagulation and their capacity to interact with DNA. Additionally, the research aimed to assess how the cross-linking process influences the structural and chemical characteristics of the composites. Detailed analyses, including microscopic examination, surface area assessment, and atomic absorption spectrometry, yielded significant results. The objective of this study was to examine the impact of calcium chloride concentration on the calcium content in alginate composites. Specifically, the study assessed how varying concentrations of the cross-linking solution (ranging from 0.5% to 2%) influence the calcium ion saturation within the composites. This investigation is essential for understanding the physicochemical properties of the materials, including calcium content, porosity, and specific surface area. The results are intended to identify the optimal cross-linking conditions that maximize calcium enrichment efficiency while preserving the material’s structural integrity. The study found that higher calcium chloride concentrations in alginate cross-linking improve the formation of a porous structure, enhanced by two-stage freeze-drying. Increased calcium levels led to a larger surface area and pore volume, and significantly higher calcium content. Furthermore, assays of activated partial thromboplastin time (aPTT) showed a reduction in clotting time for alginate composites containing calcium ions, indicating their potential as hemostatic agents. The aPTT test showed shorter clotting times with higher calcium ion concentrations, without enhanced activation of the extrinsic clotting pathway. The developed alginate material with calcium effectively supports hemostasis and reduces the risk of infection. The study also explored the capacity of these composites to interact with and modify the structure of plasmid DNA, underscoring their potential for future biomedical applications.

## 1. Introduction

Alginic materials, similar to fibers and nonwovens, are currently the focus of significant scientific interest due to their ease of production and the availability of raw materials. Additionally, they have commercial applications, which justifies continued research and development over the long term. Alginates are derived from seaweeds, primarily brown algae (*Phaeophyceae*), including species such as *Laminaria hyperborea*, *Laminaria digitata*, *Laminaria japonica*, *Ascophyllum nodosum*, and *Macrocystis pyrifera* [1,2]. Alginate is chiefly utilized as a thickening agent and stabilizer in the food, pharmaceutical, and cosmetic industries [3]. In medical applications, alginates are predominantly used in dressings designed for complex wounds [4]. Calcium alginate, a common material for wound dressings, forms a hydrophilic, non-adherent gel when it comes into contact with sodium salts found in wound exudates. The primary purpose of these dressings is to maintain a moist wound environment conducive to the healing process, due to the high absorptive capacity of alginates for wound exudates [5].

**Figure 1:**
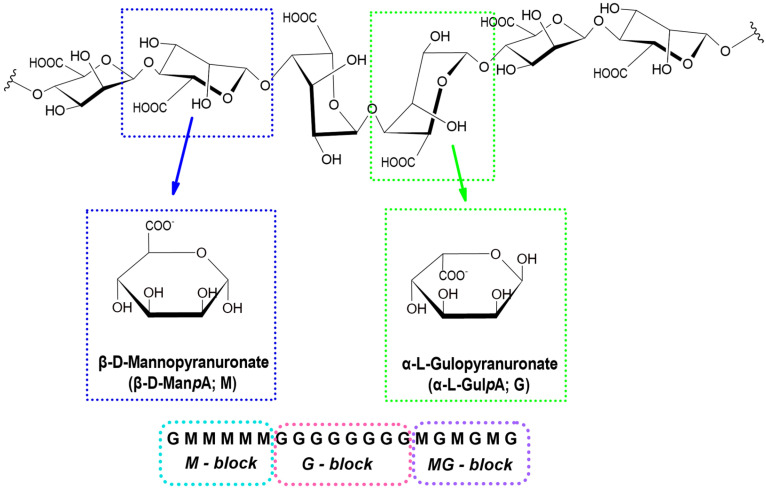
D-mannuronic and L-guluronic acid residues and types of connections of L-guluronic and D-mannuronic acid blocks in the polymer chain.

Sodium alginate, a salt of alginic acid, is extracted from the cell walls of seaweed, primarily brown algae, through a sequence of chemical and mechanical processes [4]. Alginate is a natural polysaccharide with a linear macromolecular structure. It consists of repeating blocks of β-D-mannuronic acid (M) and α-L-guluronic acid (G), linked by β-1,4-glycosidic bonds [6]. Within the macromolecules, blocks of β-D-mannuronic acid (M) and α-L-guluronic acid (G) are organized in various configurations, including MG blocks (Figure 1) [7].

Calcium alginate materials are formed through the cross-linking of sodium alginate, a chemical process driven by ion exchange. In this reaction, sodium ions (Na⁺) in sodium alginate are replaced by divalent metal ions present in the coagulation bath, such as Ca^2+^, Cu^2^⁺, Zn^2^⁺, and Ag^2+^ [2]. The formation of divalent metal alginate, which is insoluble in water, occurs through the linkage of adjacent macromolecules via primary bonds. The extent of insolubility is influenced by the substitution of sodium ions with divalent metal ions. This degree of substitution can be modulated by various factors in the cross-linking process, including the concentration of the divalent metal chloride solution, reaction time, and process temperature [8].

A new class of advanced wound dressing materials that enhance and expedite the wound healing process includes porous sponges or foams composed of polysaccharides or collagen. Alginate-based materials with foam-like porosity and distinctive properties can be synthesized using two principal methods. The first method involves creating an emulsion of an aqueous polymer solution, which is then subjected to cross-linking or foaming. The second method utilizes freezing and lyophilization of either cross-linked or non-cross-linked polymer solutions. These techniques produce a porous material with a network of interconnected pores, whose porosity and pore sizes are influenced by modifications in the technological process [9,10,11,12,13,14,15]. The simplicity of these methods, combined with low production costs and promising applications for alginate-based sponge and foam materials, has rejuvenated interest in this area.

A biodegradable alginate-based sponge with antibacterial properties designed for hemostasis of penetrating wounds was described in [16]. In this study, various molar ratios of chitosan and dopamine were combined with sodium alginate to form complexes, which were subsequently cross-linked using sodium periodate. The resulting sponge demonstrated rapid recovery after blood infusion and effectively prevented bleeding. An innovative absorbable gelatin–alginate sponge, prepared using a novel cross-linking method, was described in [17]. These sponges were enriched with active substances, including silver sulfadiazine or gentamicin sulfate, to impart antibacterial properties with a prolonged release profile of up to four days. A comprehensive range of tests, including water absorption capacity, in vitro drug release, collagenase degradation, and in vivo animal tests, were conducted to confirm the suitability of the developed material as a wound dressing. The development of a new nanocomposite sponge composed of chitosan, alginate, and carbon dots (CDs) was thoroughly detailed in [18]. These sponges were prepared using a freeze-drying method, with calcium chloride employed for cross-linking and glycerin as a plasticizer. Mass modification of the material was achieved by incorporating CDs into the sponge, resulting in versatile nanocomposite sponges for biomedical applications. The study reported that this formulation demonstrated hemostatic potential, with the in vitro blood clotting index decreasing as the CD concentration increased. The studies described in [19] demonstrated that sponges loaded with the antibiotic tetracycline hydrochloride (TCH), in combination with alginate and gelatin, exhibited significant antibacterial activity against both Gram-positive and Gram-negative bacteria. These sponges were prepared by mixing aqueous solutions of gelatin and sodium alginate, cross-linking with calcium chloride, immersing in a tetracycline hydrochloride solution, rinsing, and finally freeze-drying. The resulting samples displayed a three-dimensional network structure with high porosity, excellent swelling behavior, and controlled TCH release efficiency.

Developing an effective and safe hemostatic material that facilitates rapid blood clot formation while maintaining biocompatibility and antibacterial properties is a significant challenge for contemporary bioengineers [20]. Alginate-based biomaterials hold considerable promise in medical applications due to their advantageous characteristics, including bleeding cessation, wound healing support, biocompatibility, and biodegradability [21,22,23,24]. These alginate-based hemostatic agents are gaining popularity because of their compatibility with biological tissues and their ability to accelerate healing. They exhibit high cell viability, which enhances their safety for use in the body, and possess excellent absorbency, which enables them to effectively manage bleeding and absorb substantial amounts of bodily fluids—an essential property for treating wounds of various depths [23]. Alginate is a crucial natural polymer in the development of medical materials [25,26]. However, alginate alone does not possess intrinsic hemostatic properties [27,28].

Recent advancements in the development of alginate–calcium composites have underscored their potential as biomaterials, particularly in the fields of regenerative medicine and hemostasis. Despite these advancements, optimizing these materials for clinical applications continues to present challenges. Alginate–calcium composites, especially those synthesized via freeze-drying, exhibit promising properties such as enhanced cellular integration and efficient blood coagulation, positioning them as strong candidates for wound healing and other biomedical applications. The primary goal of this research is to build on these developments by examining the effects of alginate–calcium composites on blood clotting mechanisms, with particular attention to activated partial thromboplastin time (aPTT) and prothrombin time (PT). Additionally, this study aims to investigate the interactions between these composites and plasmid DNA through a plasmid relaxation assay, to better understand their impact on blood plasma coagulation during the initial stages of wound healing.

Furthermore, this research seeks to evaluate the potential of alginate–calcium composites as wound dressing materials by analyzing their structural features, chemical properties, and their capability to support tissue regeneration while maintaining high biocompatibility and safety. The study aims to confirm the superior efficiency and safety of these composites in comparison to currently available materials, thereby validating their suitability for future clinical applications. By optimizing the porosity of these composites, the research aims to improve cellular integration and enhance hemostatic activity, ultimately reducing clotting times and supporting tissue regeneration. Additionally, the study assesses the genotoxicity of these materials to ensure their safety for clinical use. This work offers a comprehensive analysis of alginate–calcium composites, expanding the potential applications of polysaccharide-based materials across various biomedical disciplines.

## 2. Results and Discussion

### 2.1. Methodology for the Synthesis and Fabrication of Composite Materials

**Figure 2:**
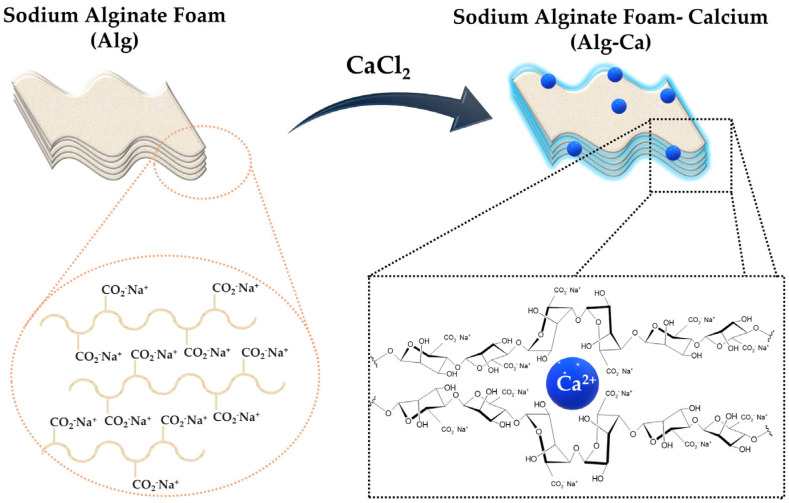
Schematic illustration of sodium alginate coagulation induced by calcium chloride (CaCl_2_).

A foam material with high porosity, derived from sodium alginate, was successfully developed utilizing the freeze-drying technique as detailed in reference [15]. The initial step involved preparing a sodium alginate solution at a concentration of 2% in water. The prepared solution was then used to create foam structures, which were subsequently subjected to a cross-linking process. This cross-linking was achieved by immersing the foams in an aqueous calcium chloride solution (Figure 2). Following the cross-linking stage, the foam samples underwent a thorough rinsing procedure with distilled water to ensure the removal of any residual chloride ions. Subsequently, the rinsed foams were frozen and subjected to freeze-drying to stabilize their porous structure.

To vary the calcium content in the final foam samples, cross-linking solutions with concentrations of 0.5%, 1%, and 2% were employed. Adjustments in cross-linking concentration are critical as they influence the extent of ionic cross-linking, which in turn affects the foam’s mechanical properties, porosity, and stability. Higher concentrations of calcium chloride enhance ionic cross-linking between alginate molecules, leading to a more robust network structure. This increase in cross-linking typically results in enhanced structural integrity and reduced pore size, potentially improving the material’s mechanical strength and its capacity for fluid absorption or retention. Conversely, lower concentrations of the cross-linking agent produce a more flexible yet less stable foam with larger pores and altered material characteristics. Additionally, the coagulation process was adjusted by varying the time (60 s) and temperature (40 °C) conditions. This approach allowed for precise control over the coagulation process, impacting the resulting material properties.

Table 1 details the coagulation conditions for alginate foams, highlighting the variations in cross-linking concentrations and their corresponding application parameters used during the foam preparation process.

The methodology outlined in detail enabled the development of a foam material with a well-structured network of interconnected pores, ranging in diameter from 0 to 359 µm. This finding validates the effectiveness of the freeze-drying technique for producing alginate-based foams with considerable porosity.

### 2.2. Analysis of Chemical and Structural Properties

#### 2.2.1. Measurement of Calcium Concentration

The calcium content in alginate samples treated with various concentrations of calcium chloride (CaCl_2_) solution during the cross-linking process was evaluated. The samples included Alg_0 (no CaCl_2_), Alg_0.5 (0.5% CaCl_2_), Alg_1 (1% CaCl_2_), and Alg_2 (2% CaCl_2_), with their respective calcium concentrations measured in mg/kg. Sample Alg_0, which was not subjected to cross-linking, has the lowest calcium concentration at 2917 mg/kg. The data indicate that the Alg_0.5 sample, treated with 0.5% CaCl_2_ solution, exhibits a calcium concentration of 41,099 mg/kg. This concentration is notably lower compared to those of the Alg_1 (1% CaCl_2_) and Alg_2 (2% CaCl_2_) samples, which have calcium concentrations of 81,830 mg/kg and 88,040 mg/kg, respectively. Interestingly, the calcium concentrations in Alg_1 and Alg_2 are comparable despite the differing concentrations of CaCl_2_, whereas Alg_0.5, treated with the lower concentration of CaCl_2_, shows a significantly reduced calcium content. These findings may be attributed to several factors associated with the alginate cross-linking process. Firstly, the calcium content in alginate is directly proportional to the concentration of CaCl_2_ used. Higher concentrations of CaCl_2_ enhance the availability of calcium ions, leading to greater incorporation into the alginate structure. Conversely, a 0.5% CaCl_2_ solution provides fewer available calcium ions, resulting in a lower calcium concentration in the alginate.

Secondly, the phenomenon of saturation might explain why the calcium concentrations in Alg_1 and Alg_2 are similar despite different CaCl_2_ concentrations. Once a critical concentration of CaCl_2_ is reached, additional increases in concentration may not proportionally enhance the calcium content in the alginate. This is due to the alginate network becoming saturated, thus limiting its ability to absorb more calcium ions linearly. Consequently, variations in CaCl_2_ concentration beyond a certain threshold may yield similar calcium concentrations in the final samples. In conclusion, the variations in calcium content in alginate, relative to CaCl_2_ concentration, reflect the balance between the availability of calcium ions and the alginate’s capacity to incorporate them, as well as the saturation effect within the alginate matrix.

Additionally, it should be noted that other parameters of the cross-linking process, such as temperature (40 °C) and duration (60 s), are kept constant across all samples. This methodology is deliberate, as our study aims to examine solely the effects of varying concentrations of CaCl_2_ on calcium concentration. Therefore, although temperature and duration are important factors in the cross-linking process, their influence is not evaluated in this research due to the consistent conditions applied throughout.

#### 2.2.2. Optical Microscopy Integrated with Elemental Analysis via Laser-Induced Breakdown Spectroscopy (LIBS)

**Figure 3:**
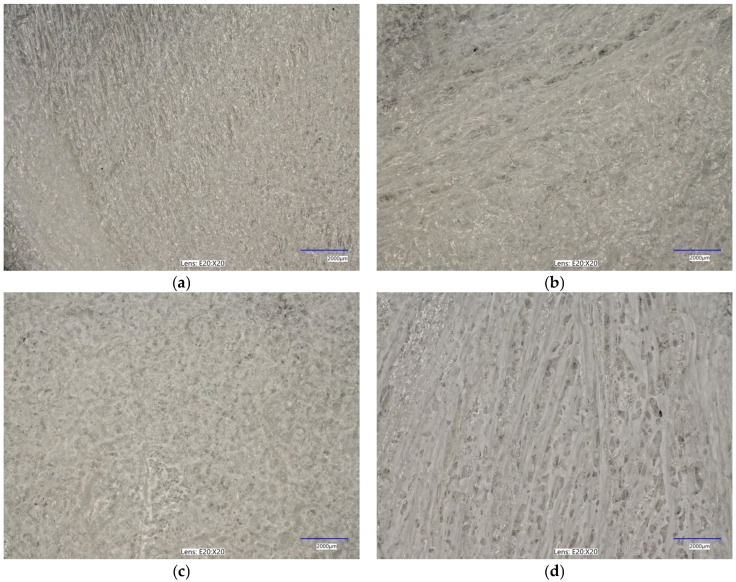
Optical microscopy images (magnifications: ×20) for the analyzed samples: (**a**) alginate (Alg_0); (**b**) Alg_0.5; (**c**) Alg_1; (**d**) Alg_2. The study was conducted through three separate and independent experimental trials. From these trials, a subset of representative results was chosen and presented for comprehensive analysis.

The immersion of alginate in a calcium chloride solution initiates the cross-linking process, during which calcium ions interact with the alginate to form a complex polymeric network. This reaction induces significant alterations in the surface morphology of the alginate, as depicted in Figure 3. Microscopic analysis of calcium-cross-linked alginate reveals the formation of a calcium ion network within the alginate structure, resulting in notable surface modifications. These changes manifest as the development of irregular nodes, as well as numerous pores and cavities. Figure 3a–d illustrate the structural and morphological transformations of the surface, which may exhibit increased roughness or wrinkling. Furthermore, the microscopic images highlight the presence of structural imperfections, such as cracks, ridges, and nodules. These surface alterations are critical for evaluating the material’s properties and potential applications in domains such as biomaterials, polymers, and composites.

**Figure 4:**
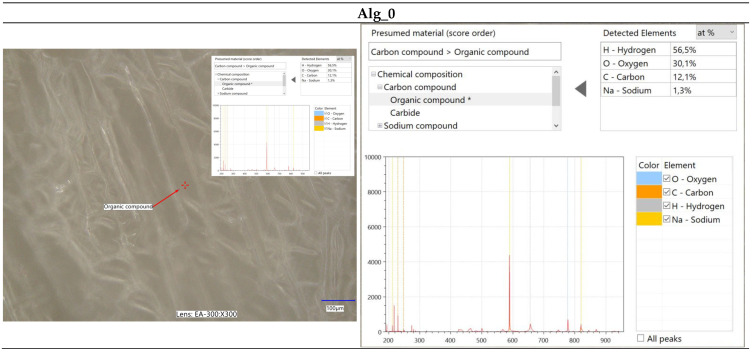
Elemental characterization of the Alg_0 sample was conducted using digital microscopy combined with laser-induced breakdown spectroscopy (LIBS).

**Figure 5:**
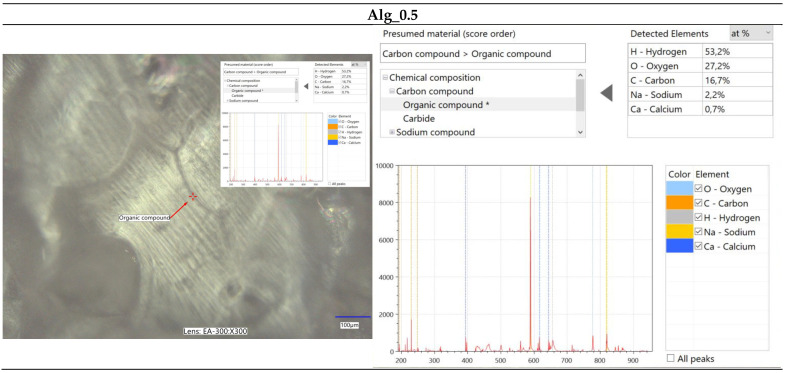
Elemental characterization of the Alg_0.5 sample was conducted using digital microscopy combined with laser-induced breakdown spectroscopy (LIBS).

**Figure 6:**
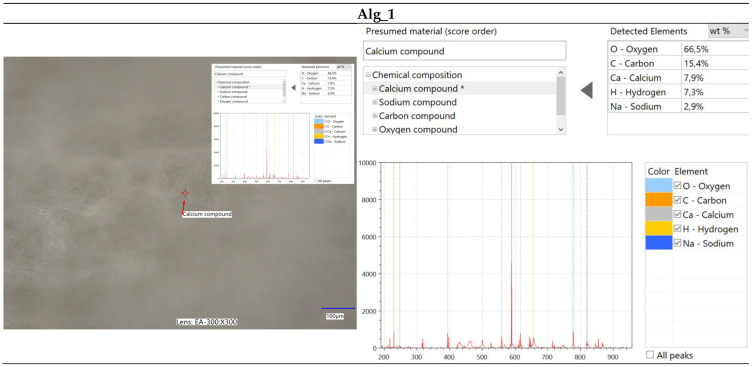
Elemental characterization of the Alg_1 sample was conducted using digital microscopy combined with laser-induced breakdown spectroscopy (LIBS).

**Figure 7:**
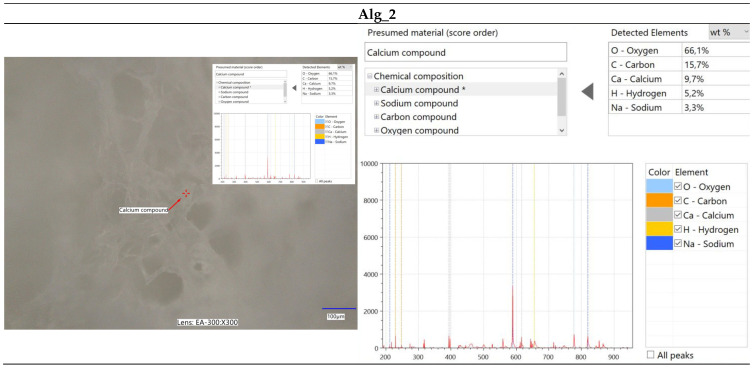
Elemental characterization of the Alg_2 sample was conducted using digital microscopy combined with laser-induced breakdown spectroscopy (LIBS).

Furthermore, spectroscopic analysis using the LIBS technique was conducted to identify and quantitatively assess the elemental composition of the samples (Figure 4, Figure 5, Figure 6 and Figure 7). The findings provided critical insights into both the chemical composition and morphological characteristics of the materials under investigation. The modification with calcium chloride (CaCl_2_) demonstrated that immersion in this solution markedly influences the distribution of elements, thereby potentially altering the physicochemical properties of the resultant material. Each stage of modification, with varying concentrations of calcium chloride, correspondingly increases the calcium content and adjusts the ratios of existing elements. This process enables the development of materials with tailored properties for specific applications.

It is noteworthy that the chemical composition analysis was conducted using laser-induced breakdown spectroscopy (LIBS), a technique that employs pulsed laser energy to determine the elemental composition of materials. While this method offers high accuracy, it is also susceptible to measurement errors that can influence the precision of the results.

#### 2.2.3. Scanning Electron Microscopy (SEM) Combined with Energy Dispersive Spectroscopy (EDS) for Elemental Analysis

A thorough comparison of scanning electron microscopy (SEM) images enables the evaluation of how calcium incorporation affects alginate morphology. By analyzing the SEM data of the samples, we can investigate surface features and identify any discernible structures within the images. At a magnification of 10,000×, which affords detailed observation of surface characteristics, we can discern variations between samples.

**Figure 8:**
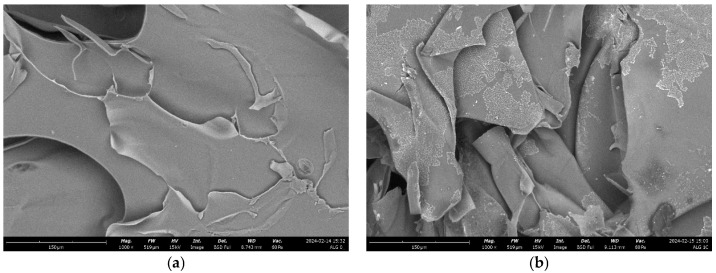

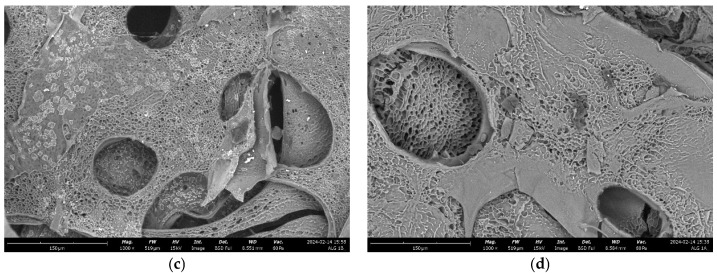
SEM results for the analyzed samples: (**a**) alginate (Alg_0); (**b**) alginate–calcium sample a (Alg_0.5); (**c**) Alg_1; (**d**) Alg_2, all observed at a magnification of 10,000×. The study was conducted through three separate and independent experimental trials. From these trials, a subset of representative results was chosen and presented for comprehensive analysis.

The scanning electron microscope (SEM) images provided illustrate clear distinctions in the microstructures of the analyzed alginate samples, especially between pure alginate (Alg_0) and alginate–calcium composites (Alg-Ca). Each image has been examined in detail. Figure 8a—alginate (Alg_0): this image depicts the pure alginate sample, which is characterized by a relatively smooth surface with minimal visible cracks. The surface maintains its integrity, with no significant porosity or irregularities, reflecting the uniformity of alginate in the absence of cross-linking agents or fillers. The first alginate–calcium composite sample (Figure 8b—Alg_0.5) demonstrates a more textured and stratified surface. The increased roughness and undulating structure can be attributed to the interaction between alginate and calcium ions, which typically induces cross-linking in alginate, resulting in a more robust and rigid structure. Despite these modifications, the surface retains a degree of coherence with observable folds and layers, showing increased density compared to pure alginate. Figure 8c—Alg_1: this image indicates a marked change in microstructure from the previous images. The surface is highly porous, with substantial voids and irregular, sponge-like structures. This porosity suggests a thorough cross-linking process, where calcium ions have formed an extensively interconnected network within the alginate matrix. The presence of large pores indicates a considerable increase in surface area, associated with greater structural stiffness and reduced flexibility. Figure 8d—Alg_2: like image (c), this sample exhibits a highly porous structure, but with even larger and more defined pores. The network here is complex, with well-defined and potentially hierarchically organized pores. This suggests a higher degree of cross-linking or a more uniform distribution of calcium ions, resulting in a distinct and highly structured appearance. The material in this state is anticipated to be extremely stiff and brittle, with minimal flexibility.

In summary, Figure 8b illustrates a more compact and layered structure, whereas Figure 8c,d display highly porous structures with substantial voids. The transition from Figure 8b to 7d signifies a progressive increase in cross-link density and material stiffness. The shift from a smooth, continuous surface in image (b) to the more porous, sponge-like structures in images (c,d) indicates a significant enhancement in cross-linking, resulting in increased rigidity and decreased flexibility compared to the structures observed in images (a,b).

##### Chemical Composition Analysis: EDS

**Figure 9:**
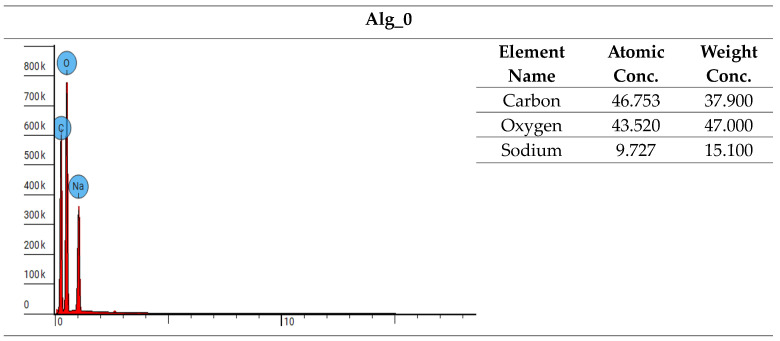

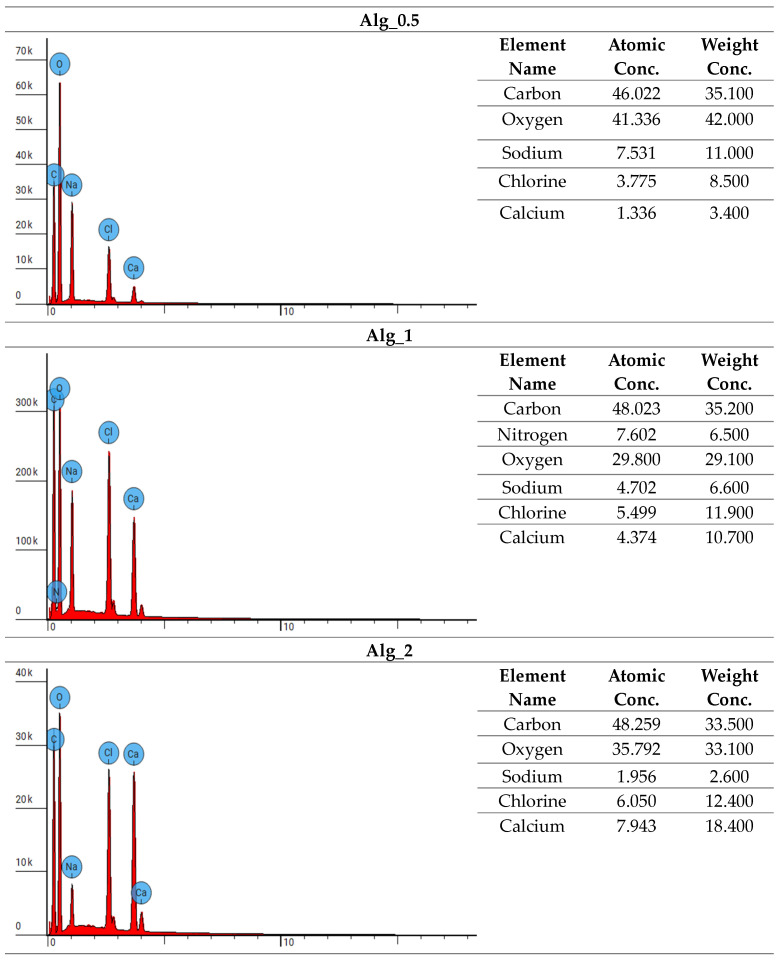
Experimental data from energy-dispersive X-ray spectroscopy (EDS). The study was conducted through three separate and independent experimental trials. From these trials, a subset of representative results was chosen and presented for comprehensive analysis.

The samples were analyzed using a scanning electron microscope equipped with energy-dispersive X-ray spectroscopy (EDS). This approach facilitated a thorough investigation of the materials’ chemical composition and allowed for precise determination of their elemental content. EDS provided detailed elemental analysis by examining the peak positions in the X-ray emission spectrum, where the intensity of the signals corresponded directly to the concentration of each element. A comprehensive summary of the analyses is presented in Figure 9.

The examination of the chemical composition for samples Alg_0, Alg_0.5, Alg_1, and Alg_2 highlights the impact of CaCl_2_ additions on elemental content. Energy-dispersive X-ray spectroscopy (EDS) analysis shows that calcium concentrations range from 3.4% in Alg_0.5 to 18.4% in Alg_2. Concurrently, the levels of carbon, sodium, and oxygen decrease, which may be attributed to chemical interactions induced by the CaCl_2_ addition. These interactions could influence solubility and alter chemical structures. The observed variations demonstrate how different concentrations of CaCl_2_ modify the chemical composition of the samples, affecting element proportions and suggesting potential chemical and structural reactions within the materials analyzed.

#### 2.2.4. Analysis of Surface Area and Total Pore Volume

The analysis of the specific surface area and total pore volume of various alginate samples—both unmodified and cross-linked with varying concentrations of calcium chloride (CaCl_2_)—provides valuable insights into their structural characteristics (Table 2). The data reveal a distinct trend: as the CaCl_2_ concentration increases, both the specific surface area and total pore volume of the samples also increase. For instance, samples cross-linked with lower CaCl_2_ concentrations, such as Alg_0.5 (0.5% CaCl_2_), exhibit lower specific surface areas and pore volumes. This is due to denser cross-linking, which results in a more compact structure with fewer and smaller pores. In contrast, samples treated with higher CaCl_2_ concentrations, such as Alg_2 (2% CaCl_2_), display significantly higher specific surface areas and pore volumes. The less dense cross-linking in these samples produces a more open structure with larger and more numerous pores.

The increase in porosity is likely attributable to the freeze-drying process, known for producing highly porous sodium alginate foams [29,30,31,32]. In this study, a two-step freeze-drying process appears critical in creating the fine-pore structures observed in the calcium alginate composites. The unmodified alginate sample (Alg_0), subjected to only a single freeze-drying step, resulted in a less porous structure. In contrast, the calcium alginate composites underwent freeze-drying both before and after cross-linking, which facilitated a more porous morphology. These findings are crucial for understanding the material properties and their potential applications, particularly in biomedical materials, polymers, and other composites where porosity significantly impacts performance. The enhanced porosity and surface area in calcium-cross-linked alginate composites suggest promising applications in fields where high surface area and porous structures are beneficial, such as drug delivery systems, tissue engineering scaffolds, and filtration materials.

**Figure 10:**
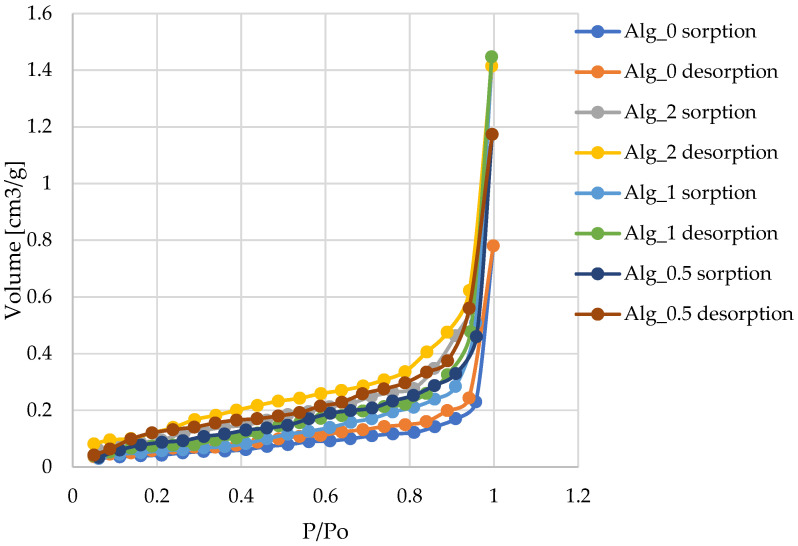
Isotherms of nitrogen adsorption–desorption at 77 K for the various materials examined in this study are presented as follows: Alg_0; Alg_0.5; Alg_1; Alg_2. The lines in the graphs represent the results of the approximation. The study was conducted through three separate and independent experimental trials. From these trials, a subset of representative results was chosen and presented for comprehensive analysis.

Analysis of the nitrogen adsorption–desorption isotherms depicted in Figure 10 reveals a pattern consistent with type III isotherms, as classified by IUPAC for physical adsorption. Such isotherms indicate minimal interactions between the adsorbent and adsorbate, resulting in the accumulation of adsorbed molecules primarily around optimal sites on the surface of non-porous or macroporous solids. The presence of type III isotherms suggests that multilayer adsorption processes are prevalent throughout the entire pressure range [33,34,35,36]. We observe an exponential increase in the quantity of adsorbate with rising pressure; however, this rate of increase is constrained, particularly at lower relative pressures. As the relative pressure approaches values close to p/p_0_ = 1, there is a marked surge in the quantity of adsorbed nitrogen. This sharp increase likely results from the diffusion of nitrogen molecules into micropores at lower pressures, followed by adsorption on the monolayer and additional layers at higher pressures [37]. Our findings indicate that such slit-shaped pores can retain some of the adsorbate even at lower pressures, which slows the initial rise in the amount of adsorbed material. Nonetheless, as the pressure increases and nears saturation, the quantity of adsorbate rises significantly, which is characteristic of slit-shaped pores.

### 2.3. Biological and Biochemical Properties

#### 2.3.1. Blood Plasma Coagulation: Activated Partial Thromboplastin Time (aPTT) and Prothrombin Time (PT)

Research indicates that divalent or multivalent cations, such as Ca^2+^, can transform soluble sodium alginate into insoluble alginate through a cross-linking process. When calcium alginate (CA) comes into contact with blood, Ca^2+^ acts as a procoagulant by activating platelets and influencing thrombin, thereby initiating the clotting cascade [38,39]. Alginate-based dressings create a naturally moist physiological environment that promotes wound healing [2,40,41,42,43].

**Figure 11:**
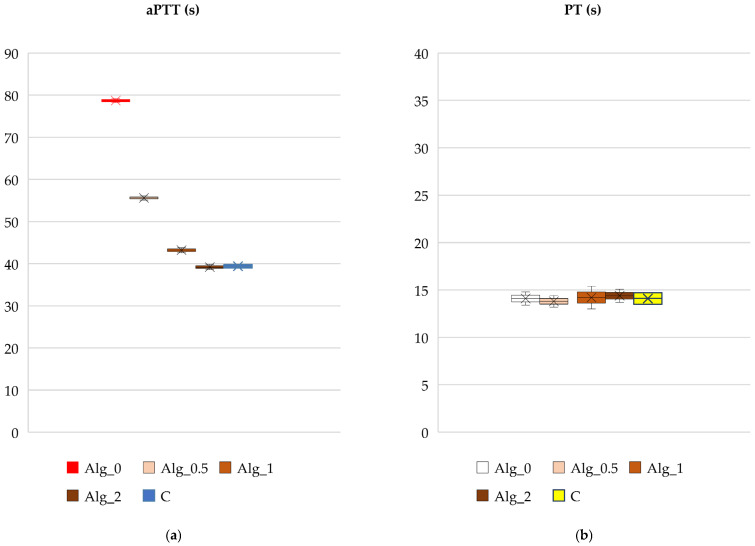
Effect of the studied alginate composites on (**a**) aPTT and (**b**) PT: Alg_0, Alg_0.5, Alg_1, Alg_2, and C (control sample: plasma not exposed to composites). Results are presented as mean (×), median (horizontal line), range (bars), and interquartile range (box).

Assessment of the compatibility of the obtained alginate materials with blood, especially with the presence of calcium ions, is crucial and therefore we analyzed them in this respect, which is important from the point of view of their potential use in medicine. In vitro tests such as partial thromboplastin time (aPTT) and prothrombin time (PT) were used to assess the antithrombogenicity of biomaterials [44,45]. Data regarding the solidification times of the obtained alginate-based materials are presented in Figure 11a,b.

The results of the activated partial thromboplastin time (aPTT), which assesses the internal coagulation mechanism through thrombin formation and fibrin clot development, indicate that the clotting times of alginate samples modified with calcium ions were shorter compared to those of unmodified samples. Specifically, as the concentration of calcium ions in the alginate samples increased, the clotting time progressively decreased. The shortest clotting time observed was for the sample with the highest calcium ion concentration (Alg_2, with a Ca^2+^ concentration of 88,040 mg/kg), as shown in Figure 11a. This reduction in clotting time is attributed to the procoagulant effect of Ca^2+^ ions.

In contrast to aPTT, prothrombin time (PT) did not exhibit any noticeable changes in response to varying calcium ion concentrations. The analysis of the data suggests that calcium levels do not significantly influence the activation of the coagulation process through the extrinsic pathway. This indicates that the extrinsic pathway’s function remains stable despite fluctuations in calcium ion concentration.

Based on these findings, we recommend the use of alginate-based hemostatic materials combined with calcium ions. These materials serve as biodegradable polymers with calcium ions functioning as coagulation activators, effectively promoting hemostasis and minimizing the risk of infection at injury sites. The insights gained from this research are relevant to various medical applications where precise bleeding control and infection minimization are critical for therapeutic efficacy. Thus, this work represents a significant advancement towards developing innovative and effective hemostatic solutions.

Activated partial thromboplastin time (aPTT), a measure of the intrinsic coagulation pathway efficiency, demonstrates a notable variation in response to increasing calcium ion concentrations. The data reveal that aPTT decreases with higher concentrations of calcium ions. For instance, the sample Alg_2 (2% CaCl_2_) exhibits the shortest aPTT at 39.17 s, whereas the sample Alg_0.5 (0.5% CaCl_2_) shows a longer aPTT of 55.60 s. This trend indicates that lower CaCl_2_ concentrations lead to a slower release of coagulation factors, thereby prolonging the aPTT. Conversely, higher CaCl_2_ concentrations, such as in Alg_2, likely create a more porous structure that facilitates a faster release and more efficient activation of coagulation factors, resulting in a shorter aPTT. This suggests that elevated CaCl_2_ levels contribute to a more compact and stable alginate foam structure, which impacts the interaction of clotting factors with the material, explaining the observed variability in aPTT.

Prothrombin time (PT), which evaluates the extrinsic clotting pathway, shows a more subtle variation with changing Ca^2+^ concentrations. For example, sample Alg_1 has the longest PT at 15.75 s, while sample Alg_0.5 displays the shortest PT at 13.80 s. The changes in PT are less pronounced compared to the variations in aPTT (Table 3).

In conclusion, the observed changes in aPTT and PT times may stem from complex interactions between CaCl_2_ and calcium alginate. The concentration of CaCl_2_ influences the structural network within alginate, potentially altering the clotting characteristics of the sample. In practical applications, varying CaCl_2_ concentrations can be leveraged to adjust the properties of alginate foams for specific purposes, such as in biomaterials or coagulation control systems. These findings underscore the necessity for further research to elucidate the impact of CaCl_2_ on coagulation mechanisms, as well as on the structure and properties of calcium alginate. A deeper understanding of these relationships could enhance the optimization of calcium alginate in diverse industrial and medical applications.

#### 2.3.2. Plasmid Relaxation Assay

**Figure 12:**
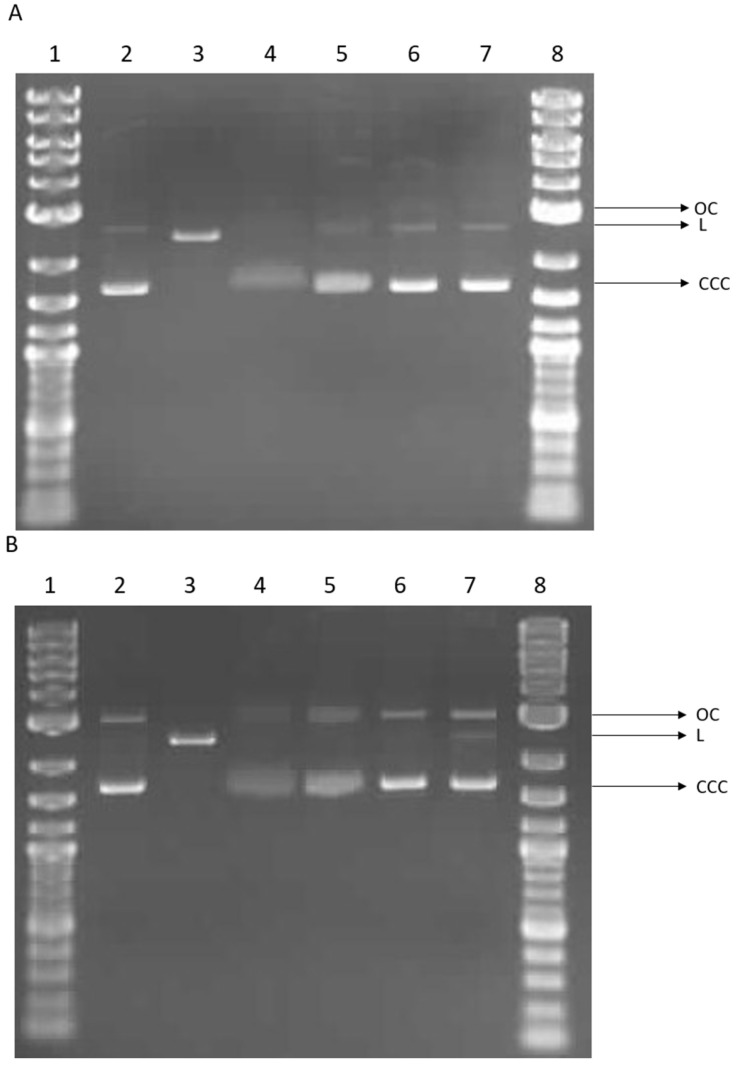
Plasmid relaxation assay. pUC19 plasmid was incubated for 2 h (**A**) and 24 h (**B**) (37 °C) with alginate (0) and alginate–calcium composites (Alg_2, Alg_1, and Alg_0.5), then was separated on a 1% agarose gel, stained with ethidium bromide, and visualized in UV light. Line 1—DNA ladder; line 2—pUC19 plasmid (the supercoiled form, CCC); line 3—pUC19 plasmid incubated with restrictase *Pst*I (the linear form, L); lines 4–7—pUC19 plasmid incubated with 0, Alg_2, Alg_1, and Alg_0.5, respectively; line 8—DNA ladder. OC—the open circular form of plasmid DNA.

We investigated the potential for direct interaction between alginate or alginate–calcium composites and DNA using a plasmid relaxation assay. Electrophoretic mobility shift analysis (EMSA) revealed that the pUC19 plasmid isolated from DH5α *E. coli* cells was predominantly in the supercoiled form (CCC). Treatment overnight at 37 °C with the restriction enzyme *Pst*I converted the plasmid to its linear form (L). Incubation of the plasmid with the 0 sample for 2 h induced structural changes in the plasmid DNA, reducing the ability of ethidium bromide to intercalate. Similar but less pronounced changes were observed with the Alg_2 sample. In contrast, incubation with Alg_1 and Alg_0.5 did not affect plasmid conformation, and the results were comparable to the control (see Figure 12A). Extended incubation (24 h) showed that the effects of the 0 sample were similar to those observed after 2 h. Additionally, the Alg_2 results were comparable to the 0 sample after extended incubation. The Alg_1 sample consistently showed results similar to the control. For the Alg_0.5 sample, the presence of the linear form (L) of the plasmid was observed (see Figure 12B).

In summary, our results indicate that alginate alters the structure of plasmid DNA and reduces the intercalation of ethidium bromide. Conversely, the presence of calcium ions appears to restore the DNA to a more complex form in a concentration-dependent manner, similar to the control. These observations suggest potential cross-linking between DNA and alginate modified by calcium. Such cross-linking could lead to DNA strand breakage and the formation of a linear plasmid form (see Figure 12B, line 7).

Various functional groups within the DNA backbone may interact with alginate, potentially introducing new physical or ionic cross-links [46]. Studies on DNA–carbon quantum dot (CQD) complexes based on alginate have demonstrated alterations in DNA mobility, with a progressive decrease in DNA gel migration observed as the weight ratio of CQDs to DNA increased [47]. Interaction of DNA with calcium ions, typically in the form of calcium chloride, results in the neutralization of the DNA’s negative charge [48]. Additionally, calcium ions facilitate the formation of electrostatic attractions within DNA molecules [49]. These properties of calcium ions contribute to the compaction of DNA, which can enhance its applicability in genetic transformation processes [50].

## 3. Materials and Methods

### 3.1. Materials

In this study, sodium alginate, a sodium salt of alginic acid derived from brown algae (Merck, Darmstadt, Germany)), with a viscosity range of 5.0 to 40.0 cps at a 2% concentration in water at 25 °C, was utilized as the base polymer for the preparation of porous polymer matrices (foams). Calcium chloride anhydrous (Merck, Darmstadt, Germany) served as the cross-linking agent, applied at three different concentrations: 0.5%, 1%, and 2%.

An aqueous solution of sodium alginate, prepared to a concentration of 2% (*w*/*v*), was mixed using a mechanical stirrer at ambient temperature until homogeneous. The solution was then allowed to stabilize for 24 h at 9 °C. For subsequent sample preparation, 100 mL of this solution was subjected to freezing at −32 °C followed by freeze-drying at −80 °C on a heated shelf set to 40 °C for a duration of 24 h. Three distinct samples were then treated with aqueous calcium chloride solutions at concentrations of 0.5%, 1%, and 2% at room temperature for 72 h. Following treatment, the foams were thoroughly rinsed with distilled water until the chlorides were completely washed out. The presence of chlorides was assessed by adding an aqueous solution of silver nitrate to the filtered rinse of the foam. Finally, the foams were dried at 40 °C until a constant mass was achieved.

### 3.2. Methods

#### 3.2.1. Measurement of Calcium Concentration

The calcium content in composite samples was determined using a single-module Magnum II microwave mineralizer (Ertec, Wrocław, Poland). Sample digestion was performed in a closed digestion system, which allowed for precise temperature and pressure control. This was achieved using the Magnum II mineralizer with the addition of 65% nitric acid (2.5 mL HNO_3_ and 2.5 mL H_2_O_2_). Quantitative analysis of calcium (Ca) was conducted using an ICP-MS 7900 (Agilent Technologies, Santa Clara, CA, USA). All measurements were conducted in duplicate, and the mean values were reported as the final results.

#### 3.2.2. Microscopic Examination—Optical and Scanning Electron Microscopy

The evaluation of surface morphology for the tested samples was conducted using both optical and scanning electron microscopy techniques. Optical microscopy was conducted using a VHX-7000N digital microscope (Keyence, Osaka, Japan) at a magnification of 20×. The elemental composition of the samples was analyzed with a digital microscope integrated with laser-induced breakdown spectroscopy (LIBS) technology. Scanning electron microscopy (SEM) analysis of the composites was carried out using a Phenom ProX G6 scanning electron microscope (Thermo Fisher Scientific, Waltham, MA, USA). SEM imaging was conducted under low vacuum conditions (60 Pa) with a probe beam energy of 15 keV. The back-scattered electron detector was employed, and magnification of 10,000× was applied. The system was equipped with an EDS X-ray microanalyzer from Oxford Instruments, located in Abingdon, UK.

#### 3.2.3. Analysis of Surface Area and Total Pore Volume

To evaluate the specific surface area and total pore volume, the Brunauer–Emmett–Teller (BET) method was employed. Measurements were conducted using an Autosorb-1 instrument (Quantachrome Instruments, Boynton Beach, FL, USA), with nitrogen (77 K) serving as the adsorption agent. Prior to analysis, the samples were dried at 105 °C for 24 h. Following this, the samples were degassed at room temperature. Approximately 2 g of each sample was weighed and utilized for the measurements.

#### 3.2.4. Blood Plasma Coagulation: Activated Partial Thromboplastin Time (aPTT) and Prothrombin Time (PT)

Human plasma that had been previously frozen and freeze-dried was dissolved in deionized water. For the assays, 1 mg of the sample was added to 200 µL of plasma. Following centrifugation, the mixture was incubated for 15 min at a controlled temperature of 37 °C. For the assessment of activated partial thromboplastin time (aPTT), a Dia-PTT reagent was prepared, which included kaolin, cephalin, and a 0.025 M calcium chloride (CaCl_2_) solution. The aPTT measurements were conducted using a K-3002 OPTIC coagulometer. Each sample involved adding 50 μL of plasma and 50 μL of the Dia-PTT suspension to the coagulometer’s thermostat, maintaining a temperature of 37 °C. After 3 min of incubation, 50 µL of the 0.025 M CaCl_2_ solution was added to initiate the measurement.

To assess prothrombin time (PT), 100 μL of the plasma sample was incubated for 2 min at a controlled temperature of 37 °C. Following this, 100 μL of the Dia-PTT suspension was added to start the measurement. The Dia-PTT suspension included rabbit brain tissue thromboplastin, calcium ions, and a preservative. To ensure the accuracy of the results, the suspension was thoroughly mixed before each use.

#### 3.2.5. Plasmid Relaxation Assay

The plasmid relaxation assay was performed based on the method described by Juszczak et al. [51]. The pUC19 plasmid was extracted from DH5α *E. coli* cells using the AxyPrep Plasmid Miniprep Kit (Axygen, Union City, CA, USA) according to the manufacturer’s protocol. The quantity and quality of the isolated plasmid DNA were assessed using the A260/A280 absorbance ratio and gel electrophoresis, respectively. The native pUC19 plasmid predominantly exists in the supercoiled conformation (CCC), which is characterized by higher electrophoretic mobility. To convert it into the linear form (L), the plasmid was treated with *Pst*I restrictase (New England Biolabs, Waltham, MA, USA). The distinct electrophoretic mobilities of the CCC and L forms are due to their topological differences.

For the assay, plasmid DNA at a concentration of 50 ng/μL was incubated with alginate (0) or alginate–calcium composites (Alg_2, Alg_1, and Alg_0.5) for 2 h and 24 h. The samples were then subjected to 1% agarose gel electrophoresis, followed by ethidium bromide staining. The stained gels were visualized under UV light (302 nm), scanned with a CCD camera, and analyzed using GeneTools software 4.1 (Syngene, Cambridge, UK). Additionally, 4 μL of a 1 kb DNA ladder (1 kb Plus DNA Ladder, New England Biolabs, Waltham, MA, USA) was run alongside the samples as a molecular weight reference.

#### 3.2.6. Statistical Analysis

Statistical evaluation of the obtained results was conducted. Data are presented as mean, standard deviation (SD), and median values. Each experiment was replicated three times. The images shown are representative of selected results.

## 4. Conclusions

In this study, the correlation between the cross-linking process parameters and the resulting chemical and structural properties of the samples was explored. The composites exhibited a finely porous structure with a considerable number of open pores. The findings suggest that increasing the concentration of the cross-linking agent during the alginate cross-linking process enhances the development of this finely porous structure in the composites. Additionally, the two-stage freeze-drying process appears to play a crucial role in promoting the formation of such a structure.

Calcium concentration analysis revealed significantly higher calcium levels in samples cross-linked with the highest concentrations of calcium chloride. These samples also exhibited the greatest specific surface area and total pore volume. The results of the activated partial thromboplastin time (aPTT) test indicated a shorter clotting time for the alginate materials containing calcium ions, with the shortest time observed at the highest calcium ion concentration, recorded at 88,040 mg/kg for sample Alg_2. The experimental data do not suggest an enhanced activation of blood clotting via the extrinsic pathway.

The findings indicate the potential of the tested composites to interact directly with DNA. Both alginate and alginate–calcium composites demonstrated the ability to alter the structure of plasmid DNA. Alginate–calcium composites likely induce DNA cross-linking, which can lead to DNA breaks. It has been shown that proper control of cross-linking process parameters allows for the production of samples with desired characteristics in terms of porosity, calcium content, and blood coagulation activity.

As a result of our work, we have developed a hemostatic material based on alginate with calcium ions, which act as blood coagulation activators and effectively ensure hemostasis while minimizing the risk of infection at the injury site.

## Figures and Tables

**Table 1:** Coagulation conditions for alginate foams using calcium chloride (CaCl_2_).

Sample Identifier	Cross-Linking Solution Concentration	Cross-Linking Temperature	Time	Composition
Alg_0	-	-	-	sodium alginate
Alg_0.5	0.5% CaCl_2_	40 °C	60 s	calcium alginate
Alg_1	1% CaCl_2_	40 °C	60 s	
Alg_2	2% CaCl_2_	40 °C	60 s	

**Table 2:** The specific surface area and total pore volume for the unmodified alginate sample and the calcium–alginate composites are analyzed. The research was carried out across three distinct and independent experimental trials.

Sample Name	Specific Surface Area			Total Pore Volume		
	Mean [m^2^/g]	Median [m^2^/g]	Standard Deviation [SD]	Mean [cm^3^/g]	Median [cm^3^/g]	Standard Deviation [SD]
Alg_0	0.3339	0.3452	0.0306	1.31 × 10^−3^	1.21 × 10^−3^	3.16 × 10^−4^
Alg_0.5	0.3770	0.3710	0.0178	1.95 × 10^−3^	1.95 × 10^−3^	1.92 × 10^−4^
Alg_1	0.4270	0.4293	0.0206	2.23 × 10^−3^	2.19 × 10^−3^	1.50 × 10^−4^
Alg_2	1.0231	1.0258	0.0201	2.38 × 10^−3^	2.24 × 10^−2^	3.02 × 10^−3^

**Table 3:** Data on aPTT and PT times for both unmodified alginate and calcium alginate composites were collected. These studies were performed across three distinct and independent experimental trials.

Sample Name	aPTT			PT		
	Mean [s]	Median [s]	Standard Deviation [SD]	Mean [s]	Median [s]	Standard Deviation [SD]
Alg_0	78.67	78.7	0.22	14.50	14.1	0.87
Alg_0.5	55.60	55.6	0.25	13.80	13.8	0.30
Alg_1	43.17	43.2	0.30	15.75	15.7	0.28
Alg_2	39.17	39.2	0.30	14.37	14.4	0.25
C	39.40	39.4	0.40	14.17	14.1	0.70

## Data Availability

The data presented in this study are available on request from the corresponding author.
